# Breastfeeding experience and anxiety in mothers with covid-19 in the postnatal period: a qualitative study

**DOI:** 10.1186/s13690-024-01285-6

**Published:** 2024-05-06

**Authors:** Handan Özcan, Tuana Güngör

**Affiliations:** 1 Department of Midwifery, University of Health Sciences Faculty of Health Sciences, Selimiye Mah. Tıbbiye Cad. No: 38, Üsküdar/İstanbul, 34668 Turkey; 2 Umraniye Training and Research Hospital, İstanbul, Turkey

**Keywords:** Covid-19, Breastfeeding, Anxiety

## Abstract

**Background:**

The fear, panic, and uncertainties arising during the Covid-19 period have caused many questions about breastfeeding. This study was conducted to investigate breastfeeding and anxiety in mothers with Covid-19.

**Methods:**

The phenomenological research type study was conducted in Istanbul between August and November 2021. The sample of the research consists of women who breastfeed after birth and who had Covid-19. Both content and descriptive analysis were used to analyze the data.

**Results:**

The data were analyzed under three main themes. Under the theme of the impact of Covid-19 on breastfeeding, mothers experienced situations like decreased or increased breastfeeding frequency, cessation of breastfeeding, isolation, anxiety about transmission, and expression of milk. They reported that their anxiety in the breastfeeding process was related to the health of the baby, baby care, decreased milk or not breastfeeding, and the Covid-19 period. They used practices such as receiving spousal and professional support, paying attention to isolation, effective communication with the baby, and praying as methods of coping with anxiety.

**Conclusion:**

The study demonstrated that factors like transmission anxiety, decreased breastfeeding frequency, and isolation affected breastfeeding, and mothers were most concerned about the baby’s health. In situations such as pandemics, protecting mother and baby health is important and a priority area. More quantitative and qualitative studies on the subject are needed.

**Table:** 

Text box 1. Contributions to the literature	
Covid-19 is an important public health problem with high morbidity and mortality rates. The contagiousness of Covid-19 is very high. When Covid 19 was identified, there was insufficient information and there were concerns about the breastfeeding process. The study aimed to determine the concerns of mothers who are breastfeeding. Findings were brought together through in-depth interviews. The study provides awareness for the management of mothers’ concerns.	

## Introduction

Covid-19 is a viral respiratory disease caused by a coronavirus that emerged in Wuhan, China towards the end of 2019. The first Covid-19 case in our country was confirmed in March 2020. In the following period, the number of cases increased in our country and the world, and the pandemic is still ongoing worldwide. Vaccination is important to prevent and protect against the spread of the virus [1]. In addition to problems in areas like public health, Covid-19 infection has also caused concerns about transmission from mother to baby during pregnancy, during childbirth, and in the postpartum period [2].

Breastfeeding during the postpartum period improves maternal and baby health and provides many social and economic benefits [3]. Breast milk positively affects the health of the baby, protects the baby against possible risks in the following periods [4], prevents situations that may adversely affect the health of the baby, and makes a valuable contribution to the prevention of possible maternal health problems [5]. ACOG (American College of Obstetricians and Gynecologists, 2021) emphasized the importance of exclusive breastfeeding for the first 6 months. Therefore, health professionals should support breastfeeding and help the mother in line with her needs, especially in the postpartum period [6].

The fear, panic, and uncertainties arising during the Covid-19 period have caused many questions about breastfeeding. Accordingly, the CDC (Centre for Disease Control) recommends that mothers confirmed or suspected of COVID-19 should wash their hands with soap and water before breastfeeding, and in the absence of soap and water, disinfectants containing at least 60% alcohol should be used. CDC also emphasizes that the mother should wear a mask during breastfeeding [1, 7]. It is reported that there is no Coronavirus infection in breast milk, thus no transmission through breastfeeding [6]. If the mother is going to express her milk, it is recommended to wear a mask and maintain hand hygiene beforehand. The milking pump and all feeding products of the baby should also be disinfected [8]. However, expressed breast milk should not be pasteurized even if the mother is Covid positive. Pasteurization reduces the immunological nutrient content of breast milk [7]. Stress, anxiety, and loneliness are also observed in cases where the mother and baby are temporarily separated due to the pandemic or social distancing measures. This may increase the risk of depression in the postpartum period [1]. UNICEF recommends that mother and baby should not be separated from each other due to COVID-19 infection, but hygiene measures should be taken to prevent transmission from mother to newborn [9].

Covid-19 is an important public health problem in terms of morbidity and mortality rates. Due to its transmission through contact and droplets, the breastfeeding process of infected mothers is particularly stressful. In the first guidelines, the mother and her baby were separated to prevent contamination, in some places the mother and her baby were prevented from contacting each other and nutrition was provided through milking, and in some places the breastfeeding process was supported without any obstruction. Especially in this confusion of information, mothers tried to manage the process with many concerns. This planned study was planned to determine the breastfeeding process of mothers who had COVID-19 infection during the breastfeeding process and the concerns they experienced during this process.

## Methods

### Participants

The study group of the research was determined with the criterion sampling method, one of the purposeful sampling methods. The criterion sampling involves people, events, objects, or situations with the qualities of the sample determined concerning the problem. Therefore, women who had Covid-19 during breastfeeding were included in the sample. The research sample consists of the snowball sampling technique, a purposive sampling method. For the data collection, 17 women living in Istanbul were interviewed. One of the women did not have enough time, and the other refused to participate in the study, so they were not included in the study. The sample size was determined based on data saturation, and the study was completed with a total of 15 participants [10].

### Data collection tools and process

The data were collected with a semi-structured interview form. The purpose of the research was explained to the participant group, they were informed that the data would be used entirely for scientific purposes, and voluntary participation of the participants was ensured. The interviews lasted an average of 30–40 min, and the mothers were interviewed over the phone or online due to the pandemic. In accordance with the basic qualitative research model, the interviews were audio recorded, and short notes were taken.

Each interview was recorded with a voice recorder and transcribed verbatim by the researcher after the interview. Inductive analysis was used to analyze the data. Inductive analysis refers to categorizing the data through coding, revealing the relationships between these categories, and creating themes and sub-themes.

The interview form created by the researchers included questions about the age, education level, occupation, obstetric history, and Covid-19 experience of the woman and her husband.

Some sample questions are below;

- Can you tell us about your previous breastfeeding process?
- Can you tell us about your breastfeeding process during the Covid-19 infection process? Have there been any changes compared to your other breastfeeding processes? Can you tell us what changes have occurred?
- Have you experienced stress and anxiety during your breastfeeding process after Covid-19 infection? Can you tell us what your concerns were?
- Can you tell us how you coped with this process?

### Inclusion criteria

- Having a Covid-19 history in the postnatal period
- Being 3–6 months postnatal
- Having a baby who is not in intensive care
- Speaking and understanding Turkish
- Agreeing to participate in the study

### Exclusion criteria

- Coordination and language problems in the woman,
- Having any psychological disorder,
- Refusing to participate in the study.

### Credibility and reliability

While presenting the data, the names were kept confdential, and each individual interviewed was coded by giving a number to facilitate the analysis (For example, P1 = Participant 1, P2 = Participant 2). Before proceeding to the analysis, the data were carefully re-read, the data remarkable for the research were coded, and categories were created by combining the appropriate codes. The themes of the study were generated by bringing together the appropriate categories. In the end, the themes were arranged and interpreted in a way that the reader can understand. Necessary criteria for validity and reliability were taken into consideration in the research. Validity includes internal and external validity, and reliability includes external and internal reliability. Internal validity refers to credibility, external validity refers to transferability, internal reliability refers to consistency, and external reliability refers to confirmation [11]. Five experts were consulted to evaluate the questionnaire prepared by the researcher to ensure credibility in the study. Necessary arrangements were made in line with the expert suggestions, and the questions were finalized. Another process for credibility was to test the comprehensibility of the questions in a pilot study with two mothers with the same characteristics before the main study. To improve reliability in this study, an international checklist (COREQ: Consolidated criteria for reporting qualitative studies) was followed during the data collection and preparation of the research report [12, 13].

### Ethics

Ethics committee approval was obtained from X University Scientific Research and Publication Ethics Committee with the date 12.07.2021 and number 46418926-050.01.04-47582.

### Statistical methods

To complete the content analysis, the data from the interviews were processed in a professional text analysis software called MAXQDA. Content analysis was used to analyze the data. Themes, codes and categories were created during the content analysis process. In the analysis of the data, the interviews recorded on the recorder were first transferred to the computer with the original data. The participants’ gestures, if any, and changes in their tone of voice were noted and the data were analyzed. While using the data, names were kept confidential and codes were given to each individual for analysis (For example, K1 for participant 1, K2 for participant 2).

The data that stood out in the analysis were coded, and categories were created by bringing together the appropriate codes. The themes of the research were created by bringing together appropriate categories. Finally, the themes were arranged and interpreted in a way that the reader could understand.

To ensure the reliability of the research, all interviews were recorded with a tape recorder and observation notes were taken by the researchers. In addition, 4 expert opinions were taken in the preparation of open-ended questions.

## Results

The mean age of the mothers and their spouses was 32.86–5.51 (min:25, max:44) and 35.60–3.69 (min:30, max:43), respectively. One of the participants (P11) had a stillbirth, and one (P5) had an abortion. Two mothers had chronic diseases (P1: Hypertension, P6: Asthma), and only four mothers (P4, P10, P11, P14) were vaccinated before getting infected with Covid-19 (Table 1).

**Table 1:** Some socio-demographic and obstetric characteristics of the mothers (n = 15)

Participant	Age	Education level	Employment status	Number of pregnancies	Number of deliveries	Number of miscarriages	Pregnancy planning status	Type of conception
P1	38	Elementary	Yes	2	1	1	Yes	Treatment
P2	25	University	No	2	1	1	Yes	Normal
P3	33	University	No	3	2	1	No	Normal
P4	29	University	No	2	1	1	Yes	Normal
P5	42	High school	No	3	2		Yes	Normal
P6	29	High school	No	2	2		No	Normal
P7	33	University	No	3	3		Yes	Normal
P8	27	High school	No	4	2	2	Yes	Normal
P9	36	Elementary	No	3	3		Yes	Normal
P10	32	Associate degree	Yes	1	1		Yes	Normal
P11	44	Elementary	No	5	4		Yes	Normal
P12	35	High school	No	2	2		Yes	Normal
P13	26	Associate degree	No	2	2		No	Normal
P14	31	University	Yes	2	1	1	Yes	Normal
P15	33	University	No	3	2	1	Yes	Normal

All the mothers in the study breastfeed their babies regularly and generally every 2 h when the baby cried day and night in positions that the baby felt comfortable.

The interview results in this study, which evaluated the breastfeeding processes Covid-19 positive mothers were discussed under 3 main themes as follows: ‘The impact of Covid-19 on breastfeeding’, ‘Covid-19 induced anxiety during breastfeeding’ and ‘Methods of coping with anxiety an stress’.

**Fig. 1:**
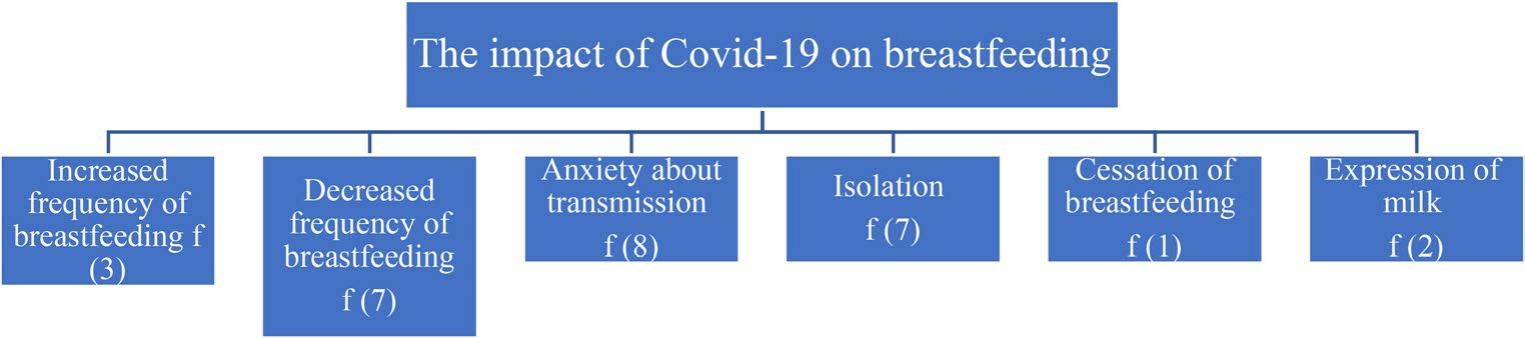
The impact of Covid-19 on the breastfeeding

In Fig. 1, six sub-themes that emerged under the impact of Covid-19 in the postpartum period on the breastfeeding process in line with the interviews with the mothers are presented. These sub-themes are increased frequency in breastfeeding, decreased frequency in breastfeeding, concern about transmission, isolation, cessation of breastfeeding, and expression of milk.

### Examples of raw data under these sub-themes are given below

Increased frequency of breastfeeding; *P1 ‘…I paid more attention to breastfeeding because my doctor told me to drink a lot of water and I did, so I breastfed more often. I mean, I never stopped, I never thought that I should not breastfeed because I was positive, I breastfed all the time’.*

#### P11

*‘There was no change in breastfeeding, even breastfeeding was more frequent. So, there was no change in the taste of the milk (laughing)’.*

Decreased frequency in breastfeeding; P8 ‘*Well, it was a little less at that time, for the first two days or so, then we went back to the routine’.*

#### P9

*‘It was a bit difficult because I was extremely nauseous, the baby wanted me, and I wanted the baby to breastfeed, but when the baby held my breast, I felt nauseous, and I wanted to vomit… You know that process exhausted me a lot. Two weeks exhausted me because I could not breastfeed my baby as comfortably as before’.*

#### P13

*‘Well, my daily breastfeeding decreased because I thought it would transmit with milk. Since I thought it would transmit with milk, my milk already decreased because I had anxiety, then I consulted my doctor, when I learned that it was not like this and it was transmitted through the respiratory tract, I started to give it by expressing it’.*

Anxiety about transmission: P2 *‘Well, since my concerns increased during the infection period, I was expressing my milk because there might be a risk of contamination to the baby, and after waiting for a while, I was giving the milk to my baby. This happened every two/two and a half hours’.*

#### P12

*‘Of course, I had a great fear because I was infected… I did not want my baby to stop breastfeeding. Therefore, I tried to continue breastfeeding in the same way as I was breastfeeding before during the infection process… I was protected with a mask, that is, in one room. I had concerns because when you become a mother, you focus directly on your child, you forget everyone else. If something happens to me and they are left behind, that was my only concern…(sad)’.*

#### P14

*‘…Of course, I was wearing my mask with the concern that I would infect my children…I tried to breastfeed with a mask and less frequently to protect them, but I still breastfed frequently’.*

Isolation: P5 *‘I used to breastfeed my baby on my lap, but after consulting our doctor, I breastfed my baby lying down instead of on my lap. I washed my hands and chest before breastfeeding and wore a double mask over my mouth and breastfed my baby that way’.*

#### P3

*‘… I continued breastfeeding by wearing my mask and not having very close one-to-one contact… I breastfed little by little and often, little by little to avoid too much contact, but I breastfed often, I did not neglect it… I preferred to breastfeed sitting down, keeping the distance, so that the less air contact the better’*

Cessation of breastfeeding: P6 *‘My milk had decreased, and I was afraid that something would happen to my baby, it would get sick, so we stopped breastfeeding’.*

Expression of milk; P10 *‘After getting infected, we switched breastfeeding to expressing milk. I was milking every two hours in the same way, we rented an electric milking machine and milked with it’.*

#### P7

*‘…because I thought that it would be transmitted with milk. My milk already decreased because I thought that it would be transmitted with milk, so I had anxiety, then I consulted my doctor, and when I learned that this was not the case, that it was transmitted through the respiratory tract, I started milking’.*

**Fig. 2:**
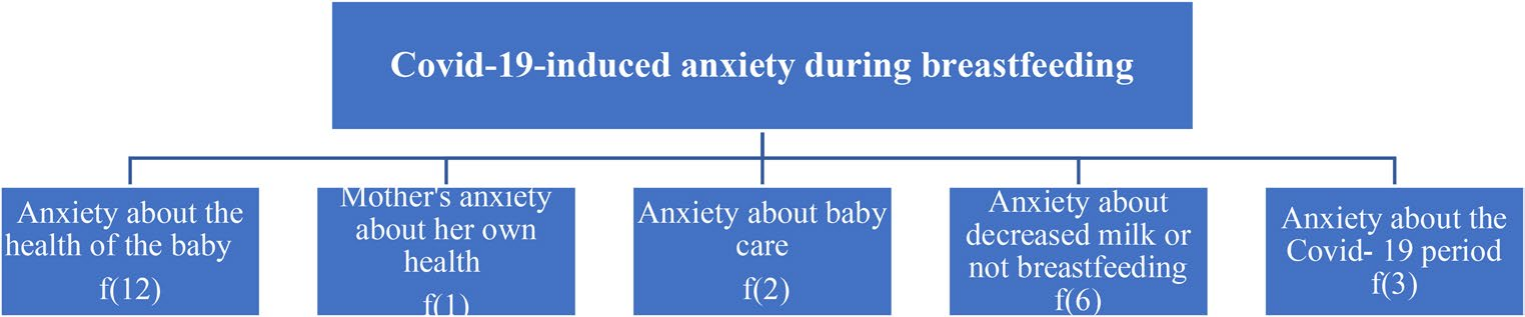
Causes of anxiety and stress due to Covid-19 experienced during breastfeeding

According to the data in the study, only one mother felt comfortable in this period and did not experience any anxiety. The causes of anxiety and stress in mothers with Covid-19 in the postpartum period were grouped under five sub-themes: anxiety about the health of the baby, mother’s anxiety about her health, anxiety about baby care, anxiety about decreased milk or not breastfeeding, and anxiety about the Covid-19 period (Fig. 2).

### Examples of raw data under these sub-themes are given below

Anxiety about the health of the baby: P7 *‘I was very afraid that the baby would get infected through breastfeeding’.*

#### P4

*‘I had a lot of anxiety (frowns). I was already worried about whether the baby would suck before birth, anyway, we got over it, then I was Covid positive, I was very worried about whether the baby would be infected, it was difficult’.*

#### P4

*‘I had a lot of anxiety (frowns). I was already worried about whether the baby would suck before birth, anyway, we got over it, then I was Covid positive, I was very worried about whether the baby would be infected, it was difficult’.*

#### P11

*‘Well, there is always stress, especially if it will harm the child’.*

Mother’s anxiety about her own health; P12 *‘I mean, I had a lot of anxiety and stress. Will I be intubated, that was always on my mind anyway’.*

Anxiety about baby care; P12 *‘My worries were mostly about who would look after my children if something happened to me. Maybe if I were single, I would not think about these things, I would probably think about other things’.*

#### P1

*‘I was worried about what would happen to my baby if I went to the hospital, who would look after him, who would give him milk, would he be deprived of breastfeeding’.*

Anxiety about decreased milk or not breastfeeding; P9 *‘…it was as if my milk decreased, I couldn’t feed, I couldn’t even drink water. I thought my milk was gone, I immediately bought formula, etc. My husband said it was psychological. The anxiety of whether the baby was full or not lasted for three or four days and I still have the formula I bought (laughs)’.*

#### P15

*‘When you have that virus, you first worry about whether you will be able to breastfeed’*.

Anxiety about the Covid- 19 period: P14 *‘I had anxiety about whether to use Covid medication. Can I use it while breastfeeding, I did not take it, but I always had a question mark in my mind, but then I said I was glad I did not take it. Everyone in the family was infected, would this process end?’.*

**Fig. 3:**
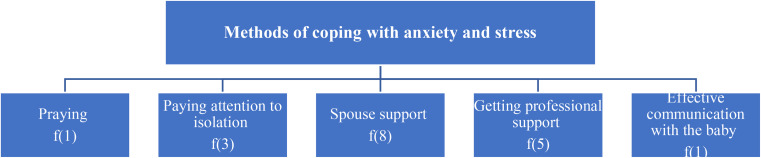
Methods of coping with anxiety and stress caused by Covid-19 experienced during breastfeeding

The methods of coping with anxiety and stress experienced by mothers with Covid-19 infection during breastfeeding were grouped under five sub-themes: praying, paying attention to isolation, spouse support, getting professional support, and effective communication with the baby (Fig. 3). Examples of raw data in these sub-themes are given below.

Praying: P1 *‘…in this process, I prayed a lot, did what my doctor said, and paid attention to isolation (thinking), so I don’t know’.*

Paying attention to isolation: P10 *‘Me and my husband wore masks, we were very careful not to infect the baby’.*

Spouse support: P2 *‘Spouse support is very important (smiles)’.*

#### P13

*‘Thanks to my husband, he helped me, otherwise, I couldn’t have done it on my own. My fever and my baby’s fever were high, and my husband was with the baby, following it and bathing it all the time’.*

Getting professional support: P4 ‘*My husband encouraged me a lot, and thanks to our midwife friend at work, she made great efforts, constantly communicated with me, and supported me’.*

#### P5

*‘They called me from the Ministry of Health and asked me to stop breastfeeding, and I was very scared and worried. Then after talking to our own doctor on the phone, we decided to continue breastfeeding, and we were relieved’.*

#### P7

*‘I was very scared at first. I was afraid that Covid would pass to the child through breastfeeding, but thank God my doctor helped me a lot, I got through the process with his support’.*

Effective communication with the baby: P3 *‘Reducing contact with the baby but still spending time with it, establishing that bond constantly, and fulfilling its needs was relaxing for me’.*

These themes are the impact of Covid-19 on breastfeeding, COVID-19-induced anxiety during breastfeeding, and methods of coping with anxiety and stress.

## Discussion

This study, conducted to examine the breastfeeding process of Covid-19 positive mothers in the postpartum period and the anxieties they experienced in this process, showed that the pandemic led to outcomes like increased breastfeeding frequency, decreased breastfeeding frequency, transmission anxiety, isolation, cessation of breastfeeding, and expression of milk. 3According to the results, during this period, mothers paid attention to isolation due to transmission, and the frequency of breastfeeding decreased. Mothers preferred to give their milk to their babies by expressing it due to the risk of transmission. Bor and colleagues (2021) found that most mothers who had Covid-19 could not breastfeed their babies during this period, and almost 90% of the mothers were concerned that the virus would pass into breast milk [14]. Mothers were encouraged to express milk during this period and were advised to do so with a special breast pump if possible. Mothers should ensure hand hygiene before each milking process, and all parts in contact with breast milk and breast should be thoroughly washed and properly disinfected after the milking process is over [15]. Maintaining uninterrupted breastfeeding by paying attention to isolation during the pandemic is important.

This study showed that mothers used different techniques to ensure the continuity of breastfeeding during Covid-19 and that they paid attention to the use of masks during breastfeeding, hand hygiene, expression of milk, and not using the expressed milk immediately. Many organizations such as WHO (World Health Organization) and CDC (Centre for Disease Control) have emphasized the importance of breast milk in the Covid-19 pandemic and the continuity of breastfeeding by applying methods such as wearing masks, paying attention to hand and breast hygiene during breastfeeding [16]. An increase in breastfeeding frequency was observed in only three mothers, and they indicated that they thought that breast milk should be increased due to infection or that they breastfed more with the recommendation of their doctors. If there is a serious COVID-19 condition in pregnant women, it seems that supporting milking is the best option to ensure the isolation of the baby and maintain milk production. Artificial milk or temporary breast milk (such as milk banking) should be used for feeding the baby. If the mother is asymptomatic, coordination between the health personnel and the mother is very important when it comes to taking the baby into the same room and breastfeeding. If the general condition of the mother is good, she can feed her baby by expressing her milk manually or with a pump. It is necessary to ensure hand hygiene and maintain contact precautions [17]. In this study, it was observed that all mothers with Covid-19 infection breastfed their babies.

Most of the women in the study experienced Covid-19-induced anxiety during breastfeeding. The most significant source of anxiety about breastfeeding in mothers during the pandemic is related to the health of the baby. Mothers had concerns about the breastfeeding period related to the decreased milk, the baby not sucking, maternal health, the care of the baby, and Covid-19. During pregnancy and postnatal periods, mothers especially experience anxiety due to fear of death, sudden illness, not being able to see, and breastfeed, and hold the baby [18]. The pandemic causes an increase in the level of anxiety in the general population. The presence of insufficient evidence, especially for the pregnancy period, exacerbates anxiety in women [19]. Separating mothers confirmed or suspected of COVID-19 from the newborn and therefore not being able to breastfeed the baby and provide skin-to-skin contact negatively affects early attachment and continuity of breastfeeding, which inevitably causes additional stress and anxiety for mothers in the postnatal periods [20]. In a case report in which postpartum conditions of Covid 19 positive pregnant women were evaluated, 2 puerperal women were discussed, and it was emphasized that the uncertainty, the mother’s inability to see, breastfeed and touch her baby increased the anxiety of women in the postpartum period [15]. Anxiety experienced during the pandemic process increases even more for sensitive or risky groups. In this study, the concerns of breastfeeding mothers are high. The anxiety experienced caused the milk supply to decrease and the baby not to be breastfed.

It is seen that especially spouse support is essential in coping with the stress experienced by the women participating in the study during the Covid-19 period. Women received professional support, paid attention to isolation and used methods such as praying or communicating about their babies. Women receiving professional support from doctors and midwives, especially during this period, is one of the effective methods to cope with their anxiety and stress. Inadequate scientific data on breastfeeding during the pandemic and misinformation on the internet and in society cause an increase in the anxiety of mothers [19]. The mothers in our study received professional support to reduce their anxiety, so healthcare professionals have a great role in accessing accurate information. The care of pregnant women diagnosed with COVID-19 includes quality and evidence-based midwifery care, a multidisciplinary team approach, maintaining isolation, reducing transmission, and providing psycho-social support to the family [21]. In addition to caring about the physical well-being of mothers, healthcare professionals should also pay attention to their mental and spiritual health and provide appropriate support for their concerns [22].

The pregnancy and postnatal periods are critical and sensitive for mothers. During breastfeeding, maternal diagnosis of Covid-19 causes anxiety. Concerns about the possibility of transmission of the infection to the baby, not being able to breastfeed her baby, being separated from her baby, and being hospitalized increase the anxiety levels of the woman. It was determined that the woman’s milk supply decreased due to the anxiety she experienced and she could not continue breastfeeding. Providing support to pregnant women, puerperal woman, and their spouses, who are a special group in situations such as pandemics, determining their anxiety levels, and providing necessary counseling is pivotal for maternal and fetal health.

## Conclusions

Breastfeeding is very important for the healthy development of a baby. The problems experienced by the mother during this period also affect breastfeeding. Especially in cases such as pandemics and disasters, the breastfeeding process is disrupted. For successful breastfeeding, it is recommended to take the necessary precautions as soon as possible and continue breastfeeding.

### Limitations of the research

The limitations of the study are that the study was conducted with a small sample and that it was conducted during the period when the covid-19 pandemic was active and information was scarce.

## Data Availability

The datasets generated during and analyzed during the current research are available from the corresponding author on reasonable request.

## References

[1] Center for Disease Control (CDC). COVID-19 Vaccine Boost Shots. 2021. https://www.cdc.gov/coronavirus/2019-ncov/vaccines/booster-shot.html (Accessed 23.01.2022).

[2] Qiao J (2020). What are the risks of COVID-19 infection in pregnant women?. Lancet.

[3] Rollins NC, Bhandari N, Hajeebhoy N, Horton S, Lutter CS, Martines JC (2016). Breastfeeding 2: why invest, and what it will take to improve breastfeeding practices. Lancet.

[4] World Health Organization (WHO). Mental Health and Psychosocial Considerations During The COVID-19 Outbreak. 2022. https://www.who.int/docs/default-source/coronaviruse/mental-health-considerations.pdf (Accessed: 07.01.2023).

[5] National Health Service. Benefits of breastfeeding 2022. https://www.nhs.uk/conditions/baby/breastfeeding-and-bottle-feeding/breastfeeding/benefits/ (Accessed 21.01.2022).

[6] Collective GB, COVID-19. Important advocacy messages about breast feeding and. 2020. https://www.globalbreastfeedingcollective.org/sites/unicef.org.globalbreastfeedingcollective/files/2020-07/Key-advocacy-messages-on-BF-and-COVID-19.pdf. (Accessed 21.01.2022).

[7] Davanzo R, Moro G, Sandri F, Agosti M, Moretti C, Mosca F (2020). Breastfeeding and coronavirus disease-2019: ad interim indications of the Italian Society of Neonatology endorsed by the Union of European Neonatal & Perinatal Societies. Matern Child Nutr.

[8] Health TCM. SARS-CoV-2 Enfeksiyonu. Rehberi, Genel Bilgiler, Epidemiyoloji ve Tanı. 2020. https://covid19.saglik.gov.tr/TR-66337/genel-bilgiler-epidemiyoloji-ve-tani.html (Accessed 19.08.2022).

[9] UNICEF. Safe breastfeeding during the COVID-19 pandemic. 2021. https://www.unicef.org/turkiye/en/stories/breastfeeding-safely-during-covid-19-pandemic. (Accessed 20.01.2022).

[10] Yılmaz H, Oral E. Menopoz.İçinde: Obstetrik Ve Jinekoloji. Demir CS, Güleç KÜ (Çeviri Editörleri). 7 ed. Baskı, Ankara, Akademisyen Tıp Kitap Evi; 2015.

[11] Erdoğan SN, Esin N, Coşansu G, Seçginli S (2014). Research in nursing.

[12] Karaçam Z (2013). Sistematik Derleme metodolojisi: Sistematik derleme hazırlamak için bir rehber. Dokuz Eylül Üniversitesi Hemşirelik Fakültesi. Elektronik Dergisi.

[13] Yıldırım A, Şimşek H. Sosyal Bilimlerinde Nitel Araştırma Yöntemleri (8. baskı). Seçkin Yayınevi, 2011.

[14] Bor NA, İpekçi NN, Öztürk M (2021). Emziren annelerin koronavirüs anksiyetesi ve emzirme öz-yeterlilikleri arasındaki ilişkinin değerlendirilmesi. Göbeklitepe Sağlık Bilimleri Dergisi.

[15] Çuvadar A, Özcan H (2020). Covid 19 enfeksiyonunda emzirme ve ebelik bakımı. Sağlık Akademisyenleri Dergisi.

[16] Tutku E, Ilıman E, Dönmez E (2020). Bireylerin Sağlik Anksiyetesi Düzeyleri İle Covid-19 Salgini Kontrol Algisinin Karşilaştirilmasi. Uluslararası Sağlık Yönetimi ve Stratejileri Araştırma. Dergisi.

[17] Wang S, Guo L, Chen L, Liu W, Cao Y, Zhang J (2020). A case report of neonatal COVID-19 infection in China. Clin Infect Dis.

[18] Liu R, Han H, Liu F (2020). Positive rate of RT-PCR detection of SARS-CoV-2 infection in 4880 cases from one hospital in Wuhan, China, from Jan to Feb 2020. Clin Chim Acta.

[19] Kınık E, Özcan H (2021). COVID-19’lu veya COVID-19 Saptanan Gebelerde Holistik Yaklaşım. Batı Karadeniz Tıp Dergisi.

[20] Chua M, Lee J, Sulaiman S, Tan H (2020). From the frontline of COVID-19-How prepared are we as obstetricians: a commentary. BJOG.

[21] Favre G, Pomar L, Qi X (2020). Guidelines for pregnant women with suspected SARS-CoV-2 infection. Lancet Infect Dis.

[22] Chen H, Guo J, Wang C, Luo F, Yu X, Zhang PW (2020). Clinical characteristics and intrauterine vertical transmission potential of COVID-19 infection in nine pregnant women: a retrospective review of medical records. Lancet.

